# Supplementary material for: Effects of Changing Veterinary Handling Techniques on Canine Behaviour and Physiology Part 1: Physiological Measurements
Source: Animals (Basel). 2023 Apr 4;13(7):1253. doi: 10.3390/ani13071253 (PMC10093362; doi:10.3390/ani13071253)
Supplement: Supplementary file 1 [file animals-13-01253-s001.zip › Figure S1 - WDQ-PET questionnaire.pdf]

VETERINARY TEACHING HOSPITAL
ATLANTIC VETERINARY COLLEGE

AVC Behavioural Medicine Service
Karen L. Overall, MA, VMD, PhD, DACVB
Camille Squair, DVM – csquair@upei.ca

In this study, we are interested in the behaviours of dogs in response to veterinary care. We will have two groups in this study and dogs will be randomly assigned to each group. We cannot blind you to the group you are in, nor can we blind the researchers, but the people evaluating the coded videos *will* be blinded as to the dog's group.

The control group will receive routine veterinary care using standard techniques. This will be a kind but standard physical exam but in a setting that does not use fear and stress reducing techniques. Venipuncture will be routine.

The interventional group will receive care using low-to-no stress and fear techniques, including those where dogs are taught to help with care.

Each group will receive some basic behaviour modification to make veterinary visits easier.

All parts of the study will be video-recorded. Videos may later be used in research, teaching and in publications, and may be used in seminars, online teaching formats, et cetera. All videos will be anonymous and humans will appear in the videos only incidentally. When they appear, their faces will be electronically blurred. The focus of the videos is simply the dog's behaviour. Dr. Camille Squair will be doing the study and Dr. Karen Overall will oversee all parts of the study

Any dog in any group can withdraw from this study at any time for any reason. After the study ends, if your dog was in the control group and there is concern that he or she is fearful or not enjoying vet visits, we are happy to schedule two more similar sessions with you (free of charge) to address this fear and to teach you the techniques we taught the first group.

If dogs in either group continue to be fearful, we will discuss interventional treatment, in depth, with your veterinarian (free of charge from us) – where ever they are – and help your veterinarian to develop an individual treatment plan.

For this study, we will meet you in the parking lot and walk you into the building. We collect data by video and ticksheet throughout. Dogs will be walked through the waiting room, weighed, taken to an exam room, given a physical exam including heart rate, respiratory rate and temperature, and a small (3-5 cc) blood sample will be taken from one of their leg veins. You will receive copies of your dog's initial full lab report at the end of the study. Dogs in the study must been seen 4 times over 2 months or every 2 weeks, and the visits will take no more than half an hour. *To participate in this study dogs must be able to eat a variety of treats including lamb, beef and/or dairy.*

Participating dogs cannot be overtly aggressive during veterinary visits since no dogs will be muzzled or undergo full physical restraint for this study. Participating dogs must be 6 months of age or older, in general good health, not pregnant, not in heat, and not taking any behavioural medications or steroids.

We seek to answer the following questions.

1. Does a standardized set of ticksheet (Likert scale) questionnaires completed by the veterinary staff represent the behaviours dogs exhibit on video during a routine veterinary examination?

2. Do patterns of behaviours that the dogs exhibit in other contexts affect or correlate with their behaviour in veterinary

exam settings?

3. Does repeated exposure to the veterinary exam setting make dogs better, worse or have no effect on their behaviour, if we control for the type and location of exposure

4. Do the behaviours exhibited by dogs during veterinary exams correlate with the physiological profile of the dogs' responses to the veterinary exam?

5. Do differences in handling style (with or without fear/stress reducing techniques) affect either behavioural or physiological responses to veterinary exams?

The generous funding for this study comes from the Sir James Dunn Animal Welfare Centre at AVC UPEI.

Question 2, above, will be answered by comparing behaviours in the exam with behaviours reported by owners in a standardized questionnaire. This questionnaire is similar to our clinical questionnaire and contains a set of questionnaires that have been validated from a series of other studies, including those involving patients and provocative testing, involving a series of researchers. Except for the questionnaire for older dogs, all other questionnaires were wholly developed by Dr. Overall, and adapted for other uses as needed.

This questionnaire is being used to screen dogs for the study involving fear in veterinary exams because we have a database of hundreds of dogs – some of whom have behavioural problems and some of whom do not – against which we can compare the results, if needed. Many of these questions were actually derived from studies on working dogs, but they do a wonderful job of characterizing how the dog responds across many situations. ***It will take you about an hour to complete this exam if your dog is active or curious and less time if the dog is fairly unfazed by the world around him or her.*** We know that this questionnaire will make you more curious about your dog and we are happy to answer your questions. If you have any questions about any aspects of this questionnaire or study, please contact Dr. Karen Overall using any of the information above (koverall@upei.ca).

Thanks so much for your participating in this important study. Please ask us if you have questions about anything. Your dog's welfare and happiness are our main concerns.

I. **Demographic Information:**

Your information (simply so that we can contact you and send you your dog's laboratory results):

1. Owner's or client's name (first and last names):	
2. Owner's or client's kennel name (if applicable):	
3. Owner's / client's address (please include country if needed to receive your lab results):	Address:<br>City:<br>Province/State:<br>Country:<br>Postal Code/Zip Code:
4. Owner's / client's Preferred phone number:	
5. Owner's / client's e-mail address:	

The rest of the questionnaire concerns information about the dog participating in this study.

1. Dog's Name (Registered name and Call name, if applicable):	
2. Does this dog have any trial placements (working dogs), awards, or titles?	☐ Yes Specify awards and trails / organizations:<br>☐ No
3. Breed of Dog:	
4. Sex of Dog:	☐ Male ☐ Female<br>☐ Castrated/Neutered ☐ Spayed<br>☐ Not Castrated/Neutered ☐ Not Spayed
5. If applicable, how old was the dog when spayed or neutered?	months old<br>☐ Unknown
6. Has this dog been bred?	☐ Yes ☐ No ☐ Unknown
7. If you have not yet bred this dog, do you plan on breeding him or her?	☐ Yes ☐ No ☐ Not sure
8. What is your dog's weight?	Kg OR Lbs
9. What colour is your dog? *(e.g. Black, Brindle, Black/Tan, etc.)*	
10. What is the EYE colour of your dog?	Left eye: Right eye:
11. What is the NOSE colour of your dog?	
12. Dog's date of birth (please estimate if unknown):	Day: Month: Year:
13. What is the source of this dog?	☐ Private show/performance dog breeder<br>☐ Private pet dog breeder<br>☐ Pet store or online purchase<br>☐ Humane shelter<br>☐ Rescue organization<br>☐ Service dog organization (e.g., CCI, Guide Dogs)<br>☐ Other (please specify):
14. At what age was your dog adopted?	weeks or months
15. Did your dog have a previous home (the breeder does **not** count as a previous home)?	☐ Yes ☐ No
16. If you answered 'Yes' to the previous question (question 15), how many other homes has this dog had?	☐ 1 ☐ 2 ☐ 3 ☐ 4 ☐ > 4 ☐ I don't know
17. When you obtained this dog, what type of training did it have?	☐ None<br>☐ Basic manners training<br>☐ Some obedience training

	☐ CGC or other titled training<br>☐ Fully trained for some job (e.g., a retired guide dog)
18. What type of training does this dog have now?	Please indicate every choice that applies:<br>☐ No school – trained yourself<br>☐ Puppy kindergarten<br>☐ Group lessons – basic<br>☐ Group lessons – advanced<br>☐ Private trainer at house<br>☐ Private trainer – sent to trainer<br>☐ Agility<br>☐ Flyball<br>☐ Nose work<br>☐ Training specifically for the AKC CGC/CKC Good Neighbour certification<br>☐ Herding<br>☐ Hunting<br>☐ Other specialty training – please specify below (e.g., stock guarding, SAR, tracking, et cetera):
19. How is the dog maintained at home? (please choose all that apply)	☐ Indoors – loose<br>☐ Indoors – crate/kennel<br>☐ Outdoors – yard<br>☐ Outdoors – run/kennel<br>☐ Outdoors – loose (unsupervised)<br>☐ Outdoors – loose (supervised)
20. Does your dog have dewclaws (thumbs on the front feet)?	☐ Yes ☐ No
21. For the study, what type of treat would your dog find most desirable (you can select multiple if applicable)	☐ Philadelphia Cream Cheese<br>☐ JIF Peanut Butter<br>☐ Kong ® Easy Spray Treat – Liver flavour<br>☐ Kong® Easy Spray Treat – Bacon and Cheese<br>☐ Mozzarella String Cheese<br>☐ Hot dogs
22. When you take your dog to the vet clinic, is your dog examined on the floor or on a table?	☐ Examined on the floor<br>☐ Examined on a table<br>☐ Unknown

End of section I of VI – there are 5 sections left to complete.

II. Reward/Reinforcement-based Questions

1. What rewards are used for correct or desired behaviours?

- ☐ N/A – I use no rewards
- ☐ Food (describe):
- ☐ Toy (describe):
- ☐ Clicker/click
- ☐ Praise
- ☐ Other (describe):

2. ***If you are holding a toy or food in front of you and at chest level*** so that **your** dog can see toy or food, how long will the dog look at or 'focus' on the toy or food without looking away at other things ongoing in the environment, **IF YOU ARE…**

a. **STANDING QUIETLY?**

- ☐ The dog does not look at the toy or food
- ☐ No more than a few seconds
- ☐ No more than 10 seconds
- ☐ No more than 30 seconds
- ☐ No more than 1 minute
- ☐ Longer than a minute
- ☐ The dog will repeatedly try and take the reward, regardless of time
- ☐ I don't know or am uncertain

b. **SQUATTING QUIETLY?**

- ☐ The dog does not look at the toy or food
- ☐ No more than a few seconds
- ☐ No more than 10 seconds
- ☐ No more than 30 seconds
- ☐ No more than 1 minute
- ☐ Longer than a minute
- ☐ I don't do this because it might be dangerous – the dog is waiting for me to throw the toy and would repeatedly try to take it
- ☐ I don't know or am uncertain

3. If you **verbally praise** your dog does he or she:

- ☐ N/A – I never verbally praise my dog
- ☐ Not look at you
- ☐ Look at you
- ☐ Look at you and cock his or her head
- ☐ Look at you and wag tail and, or body
- ☐ Look at you, cock his or her head, and wag tail and, or body
- ☐ I don't know or am uncertain.

4. ***How does your dog respond to a verbal request*** like "sit" when you do not have a treat or toy with you and **praise** is the only reward:

a. **IF YOU ARE LOOKING AT THE DOG?**

- ☐ N/A – my dog does not know the command / request
- ☐ N/A – I don't use verbal praise
- ☐ The dog does not respond
- ☐ The dog responds only after 20 sec or so and/or after repeated requests
- ☐ The dog responds after a short delay (5-10 seconds)
- ☐ The dog responds immediately or close to it.
- ☐ I don't know or am uncertain

b. **IF THE DOG CAN'T SEE YOUR FACE?**

- ☐ N/A – my dog does not know the command/request
- ☐ N/A – I don't use verbal praise
- ☐ The dog does not respond
- ☐ The dog responds only after 20 sec or so and/or after repeated requests
- ☐ The dog responds after a short delay (5-10 seconds)
- ☐ The dog responds immediately or close to it
- ☐ My dog is taught not to respond unless I am facing him or her
- ☐ I don't know or am uncertain

5. ***How does your dog respond to a verbal request like "sit" and the reward is food or a toy…***

a. **IF YOU ARE LOOKING AT THE DOG?**
- ☐ N/A – my dog does not know the command/request
- ☐ N/A – I don't use food or toys
- ☐ The dog does not respond
- ☐ The dog responds only after 20 sec or so and/or after repeated requests
- ☐ The dog responds after a short delay (5-10 seconds)
- ☐ The dog responds immediately or close to it
- ☐ I don't know or am uncertain

b. **IF THE DOG CAN'T SEE YOUR FACE?**
- ☐ N/A – my dog does not know the command/request
- ☐ N/A – I don't use food or toys
- ☐ The dog does not respond
- ☐ The dog responds only after 20 sec or so and/or after repeated requests
- ☐ The dog responds after a short delay (5-10 seconds)
- ☐ The dog responds immediately or close to it
- ☐ I don't know or am uncertain

6. How do you **"punish" or "correct"** your dog? *A punisher here is defined as an undesirable or aversive stimulus that decreases the probability of the behavior occurring again*. You may tick as many responses as are relevant.

- ☐ N/A – I don't punish or correct my dog
- ☐ I use negative punishment – I remove my attention from the dog
- ☐ I use and have a place for 'time outs'
- ☐ I calmly but firmly verbally ask / tell the dog not to do something
- ☐ I yell / scream at the dog
- ☐ I crate the dog
- ☐ I hit the dog
- ☐ I use a quick, gentle pop of a choke chain
- ☐ I use a forceful choke chain "correction"
- ☐ I apply pressure with a "pinch" collar
- ☐ I hang the dog from his collar or chain
- ☐ To achieve the desired response I choke the dog out / down
- ☐ I use an electric collar/e-collar/shock collar
- ☐ Other (please let us know what you do):

II. a. **Toy/Reward Questions (if you ONLY use food, please choose N/A)**

7. If you use **any of these items** to reward the dog do you (tick as many as are true):

a. **BALL**
- ☐ N/A – I don't use balls as rewards
- ☐ Hold it and hold on
- ☐ Throw it
- ☐ Bounce it
- ☐ Something else (specify):

b. **TOWEL/LEAD/PULL TOY**
- ☐ N/A – I don't use balls as rewards
- ☐ Hold it and hold on
- ☐ Throw it
- ☐ Bounce it
- ☐ Something else (specify):

c. **KONG® OR A BALL ON A ROPE**
- ☐ N/A – I don't use balls as rewards
- ☐ Hold it and hold on
- ☐ Throw it
- ☐ Bounce it
- ☐ Something else (specify):

8. When your dog gets his or her **reward** is he or she (*select all that apply*):

- ☐ N/A – I don't use objects as rewards
- ☐ Allowed to play alone
- ☐ Allowed to play tug with you

- [ ] Allowed to play chase with you
- [ ] Allowed to do something else – please tell us what it is:

9. If you **throw a toy** (or non-food reward object) under a vehicle or barrier does your dog:

- [ ] N/A – I have never done this
- [ ] Ignore the toy
- [ ] Look to see where the toy is and walk away
- [ ] Get down and stare at the toy
- [ ] Get down and stare at the toy and bark
- [ ] Get down and try to reach, dig, or squeeze to get to the toy **without barking**
- [ ] Get down and try to reach, dig, or squeeze to get to the toy **with barking**
- [ ] I don't know or am uncertain.

10. If your dog plays 'fetch', how does the **game usually end**?

- [ ] N/A – the dog does not play fetch
- [ ] The dog quits because he or she is interested in something else
- [ ] The dog quits because he or she is tired
- [ ] You decide when to quit
- [ ] I don't know or am uncertain.

11. Have you ever played 'fetch' with the dog **in the dark**?

- [ ] Yes
- [ ] No

End of section II of VI – there are 4 sections left to complete.

III. **Questions About Reacting to the Environment**

1. **If it is raining with *NO* THUNDER OR WIND**

☐ My dog goes out as he or she does normally
☐ My dog goes out but shortens his or her time outside
☐ My dog goes out only when he or she is desperate to eliminate
☐ I don't know or am uncertain

2. **If it is raining *WITH* THUNDER AND WIND**

☐ N/A – my dog has never heard thunder or wind
☐ My dog goes out as he or she does normally
☐ My dog goes out but shortens his or her time outside
☐ My dog goes out only when he or she is desperate to eliminate
☐ My dog goes does not go out at all
☐ I don't know or am uncertain

3. ***If it is raining or has rained*** and there are **puddles**

☐ N/A – my dog has never seen puddles
☐ My dog avoids them at all costs
☐ My dog goes through them only with encouragement
☐ My dog goes through them regardless
☐ My dog tries to play or succeeds in playing in them
☐ I don't know or am uncertain

4. ***If your dog has to walk over a new surface*** like a board placed over a hole, a subway grate, anything different, what does your dog do?

☐ My dog avoids the new surface at all costs
☐ My dog goes across the new surface only with prolonged encouragement
☐ My dog goes across the new surface with mild encouragement
☐ My dog goes across the new surface with ease and no concern
☐ I don't know or am uncertain

5. How does your dog respond if there is **a lot of continuous loud noise?**

☐ My dog attends only to the noise but stays still
☐ My dog attempts to back away from or avoid the noise
☐ My dog attends only to the noise and goes in search of it
☐ My dog listens / startles to the noise but goes back to whatever task he was engaged in, finishing more slowly
☐ My dog listens / startles to the noise but goes back to whatever task he was engaged in and finishes it as if there were no distraction
☐ There is no change in the dog's behavior
☐ I don't know or am uncertain

6. How does your dog respond if there is **intermittent loud noise?**

☐ My dog attempts to back away from or avoid the noise
☐ My dog attends only to the noise but stays still
☐ My dog attends only to the noise and goes in search of it
☐ My dog listens to the noise but goes back to whatever task he was engaged in, finishing more slowly

☐ My dog listens/startles to the noise but goes back to whatever task he was engaged in and finishes it as if there were no distraction
☐ There is no change in the dog's behavior
☐ I don't know or am uncertain

7. How does your dog respond if there **a sharp burst of noise?**

☐ My dog attempts to back away from or avoid the noise
☐ My dog attends only to the noise but stays still
☐ My dog attends only to the noise and goes in search of it
☐ My dog listens to the noise but goes back to whatever task he was engaged in, finishing more slowly
☐ My dog listens/startles to the noise but goes back to whatever task he was engaged in and finishes it as if there were no distraction
☐ There is no change in the dog's behavior
☐ I don't know or am uncertain

8. ***What does your dog do if a human approaches*** within 3 feet of your dog, seems friendly, and seeks to interact with your dog?

☐ Ignore the human
☐ Look at or watch the human but not interact
☐ Attempt to back away from or avoid the human
☐ Tremble at the sight or approach of the human
☐ Become stiff at the sight or approach of the human
☐ Crouch and, or roll at the sight of the human
☐ Interact with the human calmly, wagging tail, body
☐ Interact with the human by jumping on them
☐ I don't know or am uncertain

9. Does vocalization accompany any of the responses in question 8, above?

☐ No
☐ Yes – Please select all that apply: ☐ whines ☐ barks ☐ howls ☐ growls

10. ***What does your dog do when he or she is walking away from kennel, yard, or home another KNOWN dog approaches within 3 feet/1meter of your dog,*** seems friendly, and seeks to interact with your dog?

☐ Ignore the other dog
☐ Look at or watch the dog but not interact
☐ Attempt to back away from or avoid the dog
☐ Tremble at the sight or approach of the dog
☐ Become stiff at the sight or approach of the dog
☐ Attack the other dog
☐ Crouch and, or roll at the sight of the dog
☐ Interact with the dog calmly, wagging tail, body
☐ Interact with the dog by jumping all over them
☐ I don't know or am uncertain

11. Does vocalization accompany any of the responses in question 10, above?

☐ No
☐ Yes – Please select all that apply: ☐ whines ☐ barks ☐ howls ☐ growls

12. ***What does your dog do when he or she is walking away from kennel, yard, or home another UNKNOWN dog approaches within 3 feet/1meter of your dog***, seems friendly, and seeks to interact with your dog?

- ☐ Ignore the other dog
- ☐ Look at or watch the dog but not interact
- ☐ Attempt to back away from or avoid the dog
- ☐ Tremble at the sight or approach of the dog
- ☐ Become stiff at the sight or approach of the dog
- ☐ Attack the other dog
- ☐ Crouch and, or roll at the sight of the dog
- ☐ Interact with the dog calmly, wagging tail, body
- ☐ Interact with the dog by jumping all over them
- ☐ I don't know or am uncertain

13. Does vocalization accompany any of the responses in question 12, above?

- ☐ No
- ☐ Yes – Please select all that apply: ☐ whines ☐ barks ☐ howls ☐ growls

14. If your dog is approached by the following moving objects, how will he or she respond? Please match the response with the potential moving object in the table below by placing the relevant letters after the items in the table. There may be multiple responses to each moving object. If your dog encounters none of these items, please CHECK THE BOX

Responses:

A. Ignores the situation
B. Looks at or watches
C. Tries to withdraw
D. Tremble at the sight or approach the moving object
E. Becomes stiff at the sight or approach the moving object
F. Crouches as the moving object approaches
G. Acts calmly, wagging tail, body
H. Tries to approach the moving object chases the moving object
I. Chases the moving object
J. I don't know or am uncertain

Item	Lettered response(s) from list above:	Does vocalization accompany response?
1. Scooters<br>Does not encounter ☐		☐ No ☐ Yes<br>If yes, select all that apply:<br>☐ whines ☐ barks ☐ howls ☐ growls
2. Wheelchairs<br>Does not encounter ☐		☐ No ☐ Yes<br>If yes, select all that apply:<br>☐ whines ☐ barks ☐ howls ☐ growls
3. Baggage carts<br>Does not encounter ☐		☐ No ☐ Yes<br>If yes, select all that apply:<br>☐ whines ☐ barks ☐ howls ☐ growls
4. Motorcycles in motion<br>Does not encounter ☐		☐ No ☐ Yes<br>If yes, select all that apply:<br>☐ whines ☐ barks ☐ howls ☐ growls
5. Bicycles in motion<br>Does not encounter ☐		☐ No ☐ Yes<br>If yes, select all that apply:<br>☐ whines ☐ barks ☐ howls ☐ growls

15. If you are simply out walking the dog, how does the dog respond to **events or activities occurring** around the dog?

☐ The dog ignores everything
☐ The dog only pays active attention (in a positive way) if the event involves a lot of activity
☐ The dog pays active attention (in a positive way) to most events
☐ The dog becomes more active, curious and, or outgoing, the more that is ongoing around him or her
☐ The dog is concerned or worried, but only if the event involves a lot of activity
☐ The dog is concerned or worried with most events
☐ The dog becomes more agitated and concerned or worried, the more that is ongoing around him or her
☐ I don't know or am uncertain

16. If you think your dog becomes "worried" or "concerned", how do you know? What behaviors do you use that tell you this (check all that apply)?

☐ General body posture
☐ Tail position and activity
☐ Ear position
☐ Eye signals
☐ Vocalizations
☐ Changes in pelage or hair coat
☐ Tongue and mouth activity
☐ Overall activity

17. For your own dog and in your own words, using the information provided above, please tell us what your dogs does when worried or concerned.

18. If you are out walking with your dog, does your dog **sniff things** he or she encounters on the walk?

☐ Sometimes
☐ Almost always sniffs things in the environment, paying different amounts of attention to different objects
☐ Always sniffs things in the environment to the point where you are always dragging the dog away from one object after another.
☐ I don't know or am uncertain.

19. Below is a list of surfaces or substrates on which dogs often walk and their potential responses to those surfaces. Please check *the choice that best represents* your dog's behavior on that surface.

**Surface**	**Runs across the surface, regardless**	**Walks confidently without hesitation**	**Walks confidently but more slowly than usual**	**Walks very hesitantly**	**Won't walk across the surface**	**Dog has no experience with this type of surface**
1. Grass	☐	☐	☐	☐	☐	☐
2. Sidewalks	☐	☐	☐	☐	☐	☐
3. Blacktop roads or parking lots	☐	☐	☐	☐	☐	☐
4. Boggy ground/soil	☐	☐	☐	☐	☐	☐
5. Ground with dense vegetation	☐	☐	☐	☐	☐	☐
6. Sand	☐	☐	☐	☐	☐	☐
7. Snow	☐	☐	☐	☐	☐	☐
8. Ice	☐	☐	☐	☐	☐	☐
9. Metal surfaces	☐	☐	☐	☐	☐	☐
10. Smooth concrete surfaces	☐	☐	☐	☐	☐	☐
11. Rough/textured concrete surfaces	☐	☐	☐	☐	☐	☐
12. Marble or composite floors	☐	☐	☐	☐	☐	☐
13. Wood floors	☐	☐	☐	☐	☐	☐
14. Linoleum floors	☐	☐	☐	☐	☐	☐
15. Tile floors	☐	☐	☐	☐	☐	☐
16. Rubberized floors	☐	☐	☐	☐	☐	☐
17. Wet floors of any kind	☐	☐	☐	☐	☐	☐
18. Area rugs	☐	☐	☐	☐	☐	☐
19. Wall-to-wall carpeting	☐	☐	☐	☐	☐	☐
20. Surfaces with metal grates (boats docks, bridges)	☐	☐	☐	☐	☐	☐
21. Any highly polished surface	☐	☐	☐	☐	☐	☐
22. Rocky or disrupted surfaces	☐	☐	☐	☐	☐	☐
23. Other substrate (please describe):	☐	☐	☐	☐	☐	☐

End of section III of VI – there are 3 sections left to complete.

IV. **General Behavioural Patterns Shown by the Dog**

1. Does your dog:

- ☐ Constantly seek interaction and activity no matter what you do
- ☐ Seek interaction and activity but settle when attended to or exercised
- ☐ Not seek interaction and activity but enthusiastically accept it when offered
- ☐ Not seek interaction and activity but passively accept it when offered
- ☐ Sit or lie around quietly and show no signs of being interested in interaction or activity
- ☐ I don't know or am uncertain

2. If your dog becomes excited and pants, barks and, or moves around a lot, how do you calm him or her? *Select as many choices as apply*.

- ☐ Cannot calm dog
- ☐ Put dog in crate or kennel and ignore
- ☐ Ignore
- ☐ Walk dog until calm
- ☐ Press on or massage dog until calm
- ☐ Pet or hold dog until calm
- ☐ Tell dog that it's okay and to relax
- ☐ Other(please specify):
- ☐ I don't know or am uncertain

3. How does your dog respond to food he or she finds **on the street or any floor:**

- ☐ Eats it before you even realize what's happening
- ☐ Grabs it quickly but drops it if told to do so
- ☐ Moves towards it but is physically prevented from grabbing food
- ☐ Moves towards it but is verbally prevented from grabbing food
- ☐ Ignores the food
- ☐ I don't know or am uncertain

4. ***How does your dog respond to the following types of confinement?*** Please match the lettered response to each type of confinement for questions i. through vi. *by using the drop down list to indicate the relevant letter in each space provided.*

Responses:

A. NA – the dog is never in this situation
B. the dog is always vocalizing and moving
C. the dog stays still but vocalizes
D. the dog is quiet but is always jumping around
E. the dog occasionally moves or vocalizes when stimulated (passing person, car or animal) but is quiet more frequently than not
F. the dog is almost always quiet
G. I don't know or am not sure

i. In a vehicle for up to 15 minutes?

ii. In a vehicle for up to 30 minutes?

iii. In a vehicle for up to 1 hour or more?

iv. In a run, kennel or crate for up to 15 minutes?

v. In a run, kennel or crate for up to 30 minutes?

vi. In a run, kennel, or crate for up to 1 hour or more?

5. ***If your dog is in a crate, vehicle, run or enclosure,*** what does your dog do if:

a. **Unfamiliar dog's approach**

- ☐ N/A
- ☐ Sit quietly
- ☐ Whine
- ☐ Wag tail
- ☐ Paw at crate, vehicle, run or enclosure
- ☐ Bark
- ☐ Charge the crate vehicle, run or enclosure
- ☐ Piloerect (hair stands up)
- ☐ Posture with stiff forelegs
- ☐ Other (specify):

b. **Unfamiliar human's approach**

- ☐ N/A
- ☐ Sit quietly
- ☐ Whine
- ☐ Wag tail
- ☐ Paw at crate, vehicle, run or enclosure
- ☐ Bark
- ☐ Charge the crate vehicle, run or enclosure
- ☐ Piloerect (hair stands up)
- ☐ Posture with stiff forelegs
- ☐ Other (specify):

c. **Vehicles pass by**

- ☐ N/A
- ☐ Sit quietly
- ☐ Whine
- ☐ Wag tail
- ☐ Paw at crate, vehicle, run or enclosure
- ☐ Bark
- ☐ Charge the crate vehicle, run or enclosure
- ☐ Piloerect (hair stands up)
- ☐ Posture with stiff forelegs
- ☐ Other (specify):

End of section IV of VI – there are 2 sections left to complete

V. **Husbandry Information**

1. When your dog is alone is he or she:

- ☐ Runs free or in an enclosed yard
- ☐ Kept in a run or kennel outside
- ☐ Crates outside
- ☐ Crated inside
- ☐ Kept inside in a restricted area
- ☐ Kept inside, unrestricted
- ☐ Indoor/outdoor kennel
- ☐ I don't know or am uncertain

2. When your dog is alone does he or she do any of the following behaviors *(please circle all that apply)*?

- ☐ Eliminates
- ☐ Destroys
- ☐ Chews
- ☐ Digs
- ☐ Vocalizes
- ☐ Salivates
- ☐ Sheds
- ☐ None of the above
- ☐ I don't know what my dog does when left alone

3. Does your dog ever have ***diarrhea*** after being left alone?

- ☐ Yes
- ☐ No
- ☐ I don't know or am uncertain

4. Does your dog have ***diarrhea*** if there is a change in his or her routine?

- ☐ Yes
- ☐ No
- ☐ I don't know or am uncertain

5. Does your dog regularly have ***diarrhea*** when training / working / competing?

- ☐ N/A – not working or competitive dog and/or does no formal training
- ☐ Yes
- ☐ No
- ☐ I don't know or am uncertain

6. Does your dog have ***diarrhea*** only when trained / worked / competing in certain environment?

- ☐ N/A – not working or competitive dog and/or does no formal training
- ☐ Yes – Please tell us what environments:
- ☐ No
- ☐ I don't know or am uncertain

End of section V of VI – there is 1 section left to complete

VI. **General Behavioural and Medical History Questionnaire:**

A. ***Medical Concerns***

1. Does this dog have any recurrent, sporadic or periodic bouts of ***vomiting***?

☐ Yes
☐ No

2. Does this dog have any recurrent, sporadic, or periodic bouts of ***diarrhea/loose stool***?

☐ Yes
☐ No

3. Has this dog been evaluated for any of the following physical problems? If yes, what was the outcome?

a.	Thyroidal Illness	☐ Yes	☐ No	Outcome:
b.	Addison's Disease	☐ Yes	☐ No	Outcome:
c.	Cushing's Disease	☐ Yes	☐ No	Outcome:
d.	Hip Dysplasia	☐ Yes	☐ No	Outcome:
		If yes, what evaluation method was used?		☐ OFA ☐ Penn Hip ☐ Other – please specify:
e.	Elbow dysplasia	☐ Yes	☐ No	Outcome:
f.	Hearing (BAER test)	☐ Yes	☐ No	Outcome:
g.	Vision (CERF test)	☐ Yes	☐ No	Outcome:
h.	Cardiac screen (echocardiogram)	☐ Yes	☐ No	Outcome:

4. Has your dog been DNA tested for any *genetic diseases* (eg, not the Wisdom Panel to identify breeds in mixed breeds)?

☐ Yes - Please specify which tests, testing service used and outcomes
- Tests:
- Testing Service:
- Outcomes:

☐ No

5. Does this dog have any *other physical or medical problems* – including concerns about skin, joints, digestion, reproduction, nervous system function (e.g. epilepsy, infections like Lyme disease)?

☐ Yes – if yes, please specify:
☐ No

6. Is your dog taking any *medication or supplements for any medical or behavioral problems*?

☐ Yes – if yes, please specify:
☐ No

7. What foods, including treats, is your dog fed (brand names, please)?

8. Are/were any littermates affected with any medical problems?

☐ Yes – if yes, please specify:
☐ No
☐ Don't know

9. Are/were any littermates affected with any behavioral problems?

☐ Yes – if yes, please specify:
☐ No
☐ Don't know

10. How well does this dog do with the following commands/requests:

a. Sit
☐ My dog can't do this task because of a physical problem
☐ Perfectly
☐ Ok, needs work
☐ Badly
☐ My dog is not trained or asked to do this task

b. Stay
☐ My dog can't do this task because of a physical problem
☐ Perfectly
☐ Ok, needs work
☐ Badly
☐ My dog is not trained or asked to do this task

c. Down/lie down
☐ My dog can't do this task because of a physical problem
☐ Perfectly
☐ Ok, needs work
☐ Badly
☐ My dog is not trained or asked to do this task

d. Wait
☐ My dog can't do this task because of a physical problem
☐ Perfectly
☐ Ok, needs work
☐ Badly
☐ My dog is not trained or asked to do this task

e. Heel
☐ My dog can't do this task because of a physical problem
☐ Perfectly
☐ Ok, needs work
☐ Badly
☐ My dog is not trained or asked to do this task

f. Fetch
☐ My dog can't do this task because of a physical problem
☐ Perfectly
☐ Ok, needs work

☐ Badly
☐ My dog is not trained or asked to do this task

g. Leave it/drop it
☐ My dog can't do this task because of a physical problem
☐ Perfectly
☐ Ok, needs work
☐ Badly
☐ My dog is not trained or asked to do this task

h. Take it
☐ My dog can't do this task because of a physical problem
☐ Perfectly
☐ Ok, needs work
☐ Badly
☐ My dog is not trained or asked to do this task

i. Other (please specify):
☐ My dog can't do this task because of a physical problem
☐ Perfectly
☐ Ok, needs work
☐ Badly
☐ My dog is not trained or asked to do this task

B. ***Reactions to Absences:***

The first set of these questions deals with an **"actual absence"** - the owner actually leaves the house and the dog is either alone or totally without the owner. The second set deals with **"virtual absence"** - the owner is home, but not accessible because the door is closed or the dog is barricaded in another room. The questions are the same for each, but please answer both.

- Check **NO**, if the dog does not react in the listed circumstance.
- Check **UNKNOWN**, if you don't know.
- Check **YES**, if the dog reacts. Please evaluate the extent of the reaction from the list below.
- If **YES**:
    - 100% of the time = **always**
    - < 100% of the time, but > 60 % = **more often than not**
    - 40-60% of the time = **about equally**
    - 0% of the time <40% = **less often than not**

Behaviours during an ACTUAL absence

Behaviour	Yes	Don't Know	No
1. Destructive behaviour when separated from owner	☐ 100% of the time<br>☐ <100% but >60%<br>☐ 40-60% of the time<br>☐ >0% but <40%	☐	☐
2. Urination when separated from owner.	☐ 100% of the time<br>☐ <100% but >60%<br>☐ 40-60% of the time<br>☐ >0% but <40%	☐	☐
3. Defecation when separated from owner.	☐ 100% of the time<br>☐ <100% but >60%<br>☐ 40-60% of the time<br>☐ >0% but <40%	☐	☐
4. Vocalization when separated from owner.	☐ 100% of the time<br>☐ <100% but >60%<br>☐ 40-60% of the time<br>☐ >0% but <40%	☐	☐
5. Salivation when separated from owner.	☐ 100% of the time<br>☐ <100% but >60%<br>☐ 40-60% of the time<br>☐ >0% but <40%	☐	☐
6. Pacing when separated from owner.	☐ 100% of the time<br>☐ <100% but >60%<br>☐ 40-60% of the time<br>☐ >0% but <40%	☐	☐
7. Panting when separated from owner.	☐ 100% of the time<br>☐ <100% but >60%<br>☐ 40-60% of the time<br>☐ >0% but <40%	☐	☐
8. Trembling when separated from owner.	☐ 100% of the time<br>☐ <100% but >60%<br>☐ 40-60% of the time<br>☐ >0% but <40%	☐	☐
9. If the answer is YES for any of the above responses, what is the timing of the onset of behaviours (if known)?<br>☐ Within 5 minutes<br>☐ More than 5 minutes, but less than 30 minutes			

☐ More than 30 minutes, but less than one hour<br>☐ More than 1 hour, but less than 3 hours<br>☐ Only after several hours

Behaviours during a VIRTURAL absence

Behaviour	Yes	Don't Know	No
10. Destructive behaviour when separated from owner	☐ 100% of the time<br>☐ <100% but >60%<br>☐ 40-60% of the time<br>☐ >0% but <40%	☐	☐
11. Urination when separated from owner.	☐ 100% of the time<br>☐ <100% but >60%<br>☐ 40-60% of the time<br>☐ >0% but <40%	☐	☐
12. Defecation when separated from owner.	☐ 100% of the time<br>☐ <100% but >60%<br>☐ 40-60% of the time<br>☐ >0% but <40%	☐	☐
13. Vocalization when separated from owner.	☐ 100% of the time<br>☐ <100% but >60%<br>☐ 40-60% of the time<br>☐ >0% but <40%	☐	☐
14. Salivation when separated from owner.	☐ 100% of the time<br>☐ <100% but >60%<br>☐ 40-60% of the time<br>☐ >0% but <40%	☐	☐
15. Pacing when separated from owner.	☐ 100% of the time<br>☐ <100% but >60%<br>☐ 40-60% of the time<br>☐ >0% but <40%	☐	☐
16. Panting when separated from owner.	☐ 100% of the time<br>☐ <100% but >60%<br>☐ 40-60% of the time<br>☐ >0% but <40%	☐	☐
17. Trembling when separated from owner.	☐ 100% of the time<br>☐ <100% but >60%<br>☐ 40-60% of the time<br>☐ >0% but <40%	☐	☐
18. If the answer is YES for any of the above responses, what is the timing of the onset of behaviours (if known)?<br>☐ Within 5 minutes<br>☐ More than 5 minutes, but less than 30 minutes<br>☐ More than 30 minutes, but less than one hour<br>☐ More than 1 hour, but less than 3 hours<br>☐ Only after several hours			

C. ***Reaction to Noises***

Behaviour	Yes	Don't Know	No
1. Reaction during **thunderstorms.**<br><br>Type of response – please check all that apply:<br>☐ Salivate ☐ Tremble<br>☐ Defecate ☐ Destroy<br>☐ Urinate ☐ Freeze<br>☐ Escape ☐ Will not eat food/treats<br>☐ Pant ☐ Pupil dilation<br>☐ Vocalize (bark, whine, growl, howl) ☐ Pace<br>☐ Hide	☐ 100% of the time<br>☐ <100% but >60%<br>☐ 40-60% of the time<br>☐ >0% but <40%	☐	☐
2. Reaction to **fireworks**:<br><br>Type of response – please check all that apply:<br>☐ Salivate ☐ Tremble<br>☐ Defecate ☐ Destroy<br>☐ Urinate ☐ Freeze<br>☐ Escape ☐ Will not eat food/treats<br>☐ Pant ☐ Pupil dilation<br>☐ Vocalize (bark, whine, growl, howl) ☐ Pace<br>☐ Hide	☐ 100% of the time<br>☐ <100% but >60%<br>☐ 40-60% of the time<br>☐ >0% but <40%	☐	☐
3. Reaction to **gunshots**<br><br>Type of response – please check all that apply:<br>☐ Salivate ☐ Tremble<br>☐ Defecate ☐ Destroy<br>☐ Urinate ☐ Freeze<br>☐ Escape ☐ Will not eat food/treats<br>☐ Pant ☐ Pupil dilation<br>☐ Vocalize (bark, whine, growl, howl) ☐ Pace<br>☐ Hide	☐ 100% of the time<br>☐ <100% but >60%<br>☐ 40-60% of the time<br>☐ >0% but <40%	☐	☐
4. Reaction to **other noises**<br><br>Type(s) of noise(s) (vacuum cleaners, leaf blowers, weed whackers, dump trucks, sirens, alarm systems, etc.):<br><br>Type of response – please check all that apply:<br>☐ Salivate ☐ Tremble<br>☐ Defecate ☐ Destroy<br>☐ Urinate ☐ Freeze<br>☐ Escape ☐ Will not eat food/treats<br>☐ Pant ☐ Pupil dilation<br>☐ Vocalize (bark, whine, growl, howl) ☐ Pace<br>☐ Hide	☐ 100% of the time<br>☐ <100% but >60%<br>☐ 40-60% of the time<br>☐ >0% but <40%	☐	☐

5. How frequently in terms of *weeks* do noise events such as thunder, fireworks, or gunshots occur in the dog's environment?			
☐ Never – 0%	☐ Occasionally – >0% but <50% (once a month or so)	☐ Regularly – 50% but <100% (a few times a month)	☐ frequently – 100% (at least multiple times a week)
6. Has this dog ever been treated for noise sensitivities or phobias? If so, with what, please?			
7. Does your dog react to other aspects of storms?			
a. Wind	☐ Yes	☐ No	☐ Uncertain
b. Darkness	☐ Yes	☐ No	☐ Uncertain
c. Ozone	☐ Yes	☐ No	☐ Uncertain
d. Barometric pressure	☐ Yes	☐ No	☐ Uncertain
e. Rain	☐ Yes	☐ No	☐ Uncertain

D. ***Behaviors associated with potentially aggressive responses***

This part of the questionnaire assesses responses that are often associated with aggression or assertiveness to various situations (stimuli). **Please do not 'test' the dog to see if he or she will exhibit a response**… just tell us what you have observed in your dog's daily life.

Please read through each item and check the relevant column.

The choices for the tick sheet answers are as follow:
- **NR** = no reaction
- **S** = snarl (noise)
- **L** = lift lip (can see corner teeth)
- **B** = bark (aggressive, **not** an alerting bark)
- **G** = growl (**not** a play growl)
- **SP** = snap (no connection with skin)
- **BT** = bite (connects with skin, regardless of damage)
- **WD** = withdraw or avoid
- **NA** = not applicable (animal has never been in that situation)
- **TR** = ***this is a special category for working dogs;*** *if the dog* ***is taught to react****, check TR*

Table 1.

	NR	S	L	B	G	SP	BT	WD	NA	TR
1. Take dog's food dish with food	☐	☐	☐	☐	☐	☐	☐	☐	☐	☐
2. Take dogs empty food dish	☐	☐	☐	☐	☐	☐	☐	☐	☐	☐
3. Take dog's water dish	☐	☐	☐	☐	☐	☐	☐	☐	☐	☐
4. Take food (human) that falls on floor	☐	☐	☐	☐	☐	☐	☐	☐	☐	☐
5. Take rawhide	☐	☐	☐	☐	☐	☐	☐	☐	☐	☐
6. Take real bone	☐	☐	☐	☐	☐	☐	☐	☐	☐	☐
7. Take biscuit	☐	☐	☐	☐	☐	☐	☐	☐	☐	☐
8. Take toy	☐	☐	☐	☐	☐	☐	☐	☐	☐	☐
9. Human approaches dog while eating	☐	☐	☐	☐	☐	☐	☐	☐	☐	☐
10. Dog approaches dog while eating	☐	☐	☐	☐	☐	☐	☐	☐	☐	☐
11. Human approaches dog while playing with toys	☐	☐	☐	☐	☐	☐	☐	☐	☐	☐
12. Dog approaches dog while playing with toys	☐	☐	☐	☐	☐	☐	☐	☐	☐	☐
13. Human approaches/disturbs dog while sleeping	☐	☐	☐	☐	☐	☐	☐	☐	☐	☐
14. Dog approaches/disturbs dog while sleeping	☐	☐	☐	☐	☐	☐	☐	☐	☐	☐
15. Step over dog	☐	☐	☐	☐	☐	☐	☐	☐	☐	☐

	NR	S	L	B	G	SP	BT	WD	NA	TR
16. Push dog off bed/couch	☐	☐	☐	☐	☐	☐	☐	☐	☐	☐
17. Reach toward dog	☐	☐	☐	☐	☐	☐	☐	☐	☐	☐
18. Reach over head	☐	☐	☐	☐	☐	☐	☐	☐	☐	☐
19. Put on leash	☐	☐	☐	☐	☐	☐	☐	☐	☐	☐
20. Push on shoulders	☐	☐	☐	☐	☐	☐	☐	☐	☐	☐
21. Push on rump	☐	☐	☐	☐	☐	☐	☐	☐	☐	☐
22. Towel feet when wet	☐	☐	☐	☐	☐	☐	☐	☐	☐	☐
23. Bathe dog	☐	☐	☐	☐	☐	☐	☐	☐	☐	☐
24. Groom dog’s head	☐	☐	☐	☐	☐	☐	☐	☐	☐	☐
25. Groom dog’s body	☐	☐	☐	☐	☐	☐	☐	☐	☐	☐
26. Stare at	☐	☐	☐	☐	☐	☐	☐	☐	☐	☐
27. Take muzzle in hands and shake	☐	☐	☐	☐	☐	☐	☐	☐	☐	☐
28. Push dog over onto back	☐	☐	☐	☐	☐	☐	☐	☐	☐	☐
29. Stranger knocks on door	☐	☐	☐	☐	☐	☐	☐	☐	☐	☐
30. Stranger enters room	☐	☐	☐	☐	☐	☐	☐	☐	☐	☐
31. Dog in car at toll booth	☐	☐	☐	☐	☐	☐	☐	☐	☐	☐
32. Dog in car at gas station	☐	☐	☐	☐	☐	☐	☐	☐	☐	☐
33. Dog on leash approached by known dog on street	☐	☐	☐	☐	☐	☐	☐	☐	☐	☐
34. Dog on leash approached by unknown dog on street	☐	☐	☐	☐	☐	☐	☐	☐	☐	☐
35. Dog on leash approached by person on street	☐	☐	☐	☐	☐	☐	☐	☐	☐	☐
36. Dog in yard – person passes	☐	☐	☐	☐	☐	☐	☐	☐	☐	☐
37. Dog in yard – unknown dog passes	☐	☐	☐	☐	☐	☐	☐	☐	☐	☐
38. Dog in yard – known dog passes	☐	☐	☐	☐	☐	☐	☐	☐	☐	☐
39. Dog in vet’s office	☐	☐	☐	☐	☐	☐	☐	☐	☐	☐
40. Dog in boarding kennel	☐	☐	☐	☐	☐	☐	☐	☐	☐	☐

	NR	S	L	B	G	SP	BT	WD	NA	TR
41. Dog in groomers	☐	☐	☐	☐	☐	☐	☐	☐	☐	☐
42. Dog yelled at	☐	☐	☐	☐	☐	☐	☐	☐	☐	☐
43. Dog corrected with leash	☐	☐	☐	☐	☐	☐	☐	☐	☐	☐
44. Dog physical punished – hit	☐	☐	☐	☐	☐	☐	☐	☐	☐	☐
45. Someone raised voice to owner in presence of dog	☐	☐	☐	☐	☐	☐	☐	☐	☐	☐
46. Someone hugs/touches owner in presence of dog	☐	☐	☐	☐	☐	☐	☐	☐	☐	☐
47. Squirrels, cats, small animals' approach	☐	☐	☐	☐	☐	☐	☐	☐	☐	☐
48. Bicycles, skateboards	☐	☐	☐	☐	☐	☐	☐	☐	☐	☐
49. Crying infant	☐	☐	☐	☐	☐	☐	☐	☐	☐	☐
50. Playing with 2-year-old children	☐	☐	☐	☐	☐	☐	☐	☐	☐	☐
51. Playing with 5-7-year-old children	☐	☐	☐	☐	☐	☐	☐	☐	☐	☐
52. Playing with 8-11-year-old children	☐	☐	☐	☐	☐	☐	☐	☐	☐	☐
53. Playing with 12-16-year-old children	☐	☐	☐	☐	☐	☐	☐	☐	☐	☐

If you chose the category "WD/withdraw/avoid" for any of the behavioural scenarios listed in table 1, please tell us which, if any, of the behaviours listed your dog also exhibits when he or she withdraws. If the dog does no additional behaviors except withdraw or avoid, please do NONE. You ONLY need to complete the question numbers for which you have already checked WD in the above questionnaire. This one should be quick to complete.

The choices for the tick sheet answers are as follow:

- **SAL** – salivate
- **DEF** – defecate
- **URI** – Urinate
- **DES** – Destroy
- **ESC** – Escape
- **VOC** – Vocalize
- **PAC** – Pace
- **FRE** – Freeze
- **PAN** – Pant
- **TRE** – Tremble
- **LOW** – Lower body posture/drop down
- **NONE** – no additional behaviours

Table 2.

	SAL	DEF	URI	DES	ESC	VOC	PAC	FRE	PAN	TRE	LOW	NONE
1. Take dog's food dish with food	☐	☐	☐	☐	☐	☐	☐	☐	☐	☐	☐	☐
2. Take dogs empty food dish	☐	☐	☐	☐	☐	☐	☐	☐	☐	☐	☐	☐
3. Take dog's water dish	☐	☐	☐	☐	☐	☐	☐	☐	☐	☐	☐	☐
4. Take food (human) that falls on floor	☐	☐	☐	☐	☐	☐	☐	☐	☐	☐	☐	☐
5. Take rawhide	☐	☐	☐	☐	☐	☐	☐	☐	☐	☐	☐	☐
6. Take real bone	☐	☐	☐	☐	☐	☐	☐	☐	☐	☐	☐	☐
7. Take biscuit	☐	☐	☐	☐	☐	☐	☐	☐	☐	☐	☐	☐
8. Take toy	☐	☐	☐	☐	☐	☐	☐	☐	☐	☐	☐	☐
9. Human approaches dog while eating	☐	☐	☐	☐	☐	☐	☐	☐	☐	☐	☐	☐
10. Dog approaches dog while eating	☐	☐	☐	☐	☐	☐	☐	☐	☐	☐	☐	☐
11. Human approaches dog while playing with toys	☐	☐	☐	☐	☐	☐	☐	☐	☐	☐	☐	☐
12. Dog approaches dog while playing with toys	☐	☐	☐	☐	☐	☐	☐	☐	☐	☐	☐	☐
13. Human approaches/disturbs dog while sleeping	☐	☐	☐	☐	☐	☐	☐	☐	☐	☐	☐	☐
14. Dog approaches/disturbs dog while sleeping	☐	☐	☐	☐	☐	☐	☐	☐	☐	☐	☐	☐
15. Step over dog	☐	☐	☐	☐	☐	☐	☐	☐	☐	☐	☐	☐
16. Push dog off bed/couch	☐	☐	☐	☐	☐	☐	☐	☐	☐	☐	☐	☐
17. Reach toward dog	☐	☐	☐	☐	☐	☐	☐	☐	☐	☐	☐	☐
18. Reach over head	☐	☐	☐	☐	☐	☐	☐	☐	☐	☐	☐	☐

	SAL	DEF	URI	DES	ESC	VOC	PAC	FRE	PAN	TRE	LOW	NONE
19. Put on leash	☐	☐	☐	☐	☐	☐	☐	☐	☐	☐	☐	☐
20. Push on shoulders	☐	☐	☐	☐	☐	☐	☐	☐	☐	☐	☐	☐
21. Push on rump	☐	☐	☐	☐	☐	☐	☐	☐	☐	☐	☐	☐
22. Towel feet when wet	☐	☐	☐	☐	☐	☐	☐	☐	☐	☐	☐	☐
23. Bathe dog	☐	☐	☐	☐	☐	☐	☐	☐	☐	☐	☐	☐
24. Groom dog’s head	☐	☐	☐	☐	☐	☐	☐	☐	☐	☐	☐	☐
25. Groom dog’s body	☐	☐	☐	☐	☐	☐	☐	☐	☐	☐	☐	☐
26. Stare at	☐	☐	☐	☐	☐	☐	☐	☐	☐	☐	☐	☐
27. Take muzzle in hands and shake	☐	☐	☐	☐	☐	☐	☐	☐	☐	☐	☐	☐
28. Push dog over onto back	☐	☐	☐	☐	☐	☐	☐	☐	☐	☐	☐	☐
29. Stranger knocks on door	☐	☐	☐	☐	☐	☐	☐	☐	☐	☐	☐	☐
30. Stranger enters room	☐	☐	☐	☐	☐	☐	☐	☐	☐	☐	☐	☐
31. Dog in car at toll booth	☐	☐	☐	☐	☐	☐	☐	☐	☐	☐	☐	☐
32. Dog in car at gas station	☐	☐	☐	☐	☐	☐	☐	☐	☐	☐	☐	☐
33. Dog on leash approached by known dog on street	☐	☐	☐	☐	☐	☐	☐	☐	☐	☐	☐	☐
34. Dog on leash approached by unknown dog on street	☐	☐	☐	☐	☐	☐	☐	☐	☐	☐	☐	☐
35. Dog on leash approached by person on street	☐	☐	☐	☐	☐	☐	☐	☐	☐	☐	☐	☐
36. Dog in yard – person passes	☐	☐	☐	☐	☐	☐	☐	☐	☐	☐	☐	☐
37. Dog in yard – unknown dog passes	☐	☐	☐	☐	☐	☐	☐	☐	☐	☐	☐	☐
38. Dog in yard – known dog passes	☐	☐	☐	☐	☐	☐	☐	☐	☐	☐	☐	☐
39. Dog in vet’s office	☐	☐	☐	☐	☐	☐	☐	☐	☐	☐	☐	☐
40. Dog in boarding kennel	☐	☐	☐	☐	☐	☐	☐	☐	☐	☐	☐	☐
41. Dog in groomers	☐	☐	☐	☐	☐	☐	☐	☐	☐	☐	☐	☐
42. Dog yelled at	☐	☐	☐	☐	☐	☐	☐	☐	☐	☐	☐	☐
43. Dog corrected with leash	☐	☐	☐	☐	☐	☐	☐	☐	☐	☐	☐	☐

	SAL	DEF	URI	DES	ESC	VOC	PAC	FRE	PAN	TRE	LOW	NONE
44. Dog physical punished – hit	☐	☐	☐	☐	☐	☐	☐	☐	☐	☐	☐	☐
45. Someone raised voice to owner in presence of dog	☐	☐	☐	☐	☐	☐	☐	☐	☐	☐	☐	☐
46. Someone hugs/touches owner in presence of dog	☐	☐	☐	☐	☐	☐	☐	☐	☐	☐	☐	☐
47. Squirrels, cats, small animals' approach	☐	☐	☐	☐	☐	☐	☐	☐	☐	☐	☐	☐
48. Bicycles, skateboards	☐	☐	☐	☐	☐	☐	☐	☐	☐	☐	☐	☐
49. Crying infant	☐	☐	☐	☐	☐	☐	☐	☐	☐	☐	☐	☐
50. Playing with 2-year-old children	☐	☐	☐	☐	☐	☐	☐	☐	☐	☐	☐	☐
51. Playing with 5-7-year-old children	☐	☐	☐	☐	☐	☐	☐	☐	☐	☐	☐	☐
52. Playing with 8-11-year-old children	☐	☐	☐	☐	☐	☐	☐	☐	☐	☐	☐	☐
53. Playing with 12-16-year-old children	☐	☐	☐	☐	☐	☐	☐	☐	☐	☐	☐	☐

E. ***Stereotypic and Ritualistic Behavior History***

Please complete this form ***ONLY*** if the dog is showing any repetitive, ritualistic behaviors. *If your dog shows none of these behaviours*, CHECK THIS BOX ☐

This section focuses on a ***description and categorization*** of the dog's behavior(s) as described by the handler.

1. Does the dog do any of the following behaviors? **Tick as many categories that apply to the dog's behaviour.** Then check the best description that relates to the selected behaviour.	
☐ Grooming	☐ Chewing self<br>☐ Licking self<br>☐ Barbering/trimming hair on self<br>☐ Sucking self<br>☐ Biting self<br>☐ Plucking hair from self<br>☐ Continuously doing any of these behaviours to *another individual*. Please elaborate:<br>☐ Other, please explain:
☐ Hallucinatory	☐ Staring and attending to things that are not there<br>☐ Tracking things that are not there<br>☐ Pouncing on or attacking things that are not there<br>☐ Other, please explain:
☐ Consumptive	☐ Consuming rocks<br>☐ Consuming dirt or soil<br>☐ Consuming other objects<br>☐ Eating, licking, sucking or chewing wool or fabric, rugs, furniture, etc.<br>☐ Licking or gulping air<br>☐ Other, please explain:
☐ Locomotory	☐ Circling / spinning<br>☐ Tail-chasing<br>☐ Freezing<br>☐ Other, please explain:
☐ Vocalization	☐ Rhythmic barking<br>☐ Howling<br>☐ Growling<br>☐ Other, please explain:

This next section focuses on ***patterns*** of behaviors.

	**Yes**	**No**	**Unknown**
1. Was there a change in the household or an event associated with the development of the behaviour?	If yes, what?	☐	☐
2. Is there any time of day when the behaviour seems more or less intense?	If yes, what time?	☐	☐
3. Have you witnessed the behaviour causing damage?		☐	☐
4. If so, can you stop the behaviour?	If yes, how:	☐	☐
5. Does any event or behaviour routinely occur immediately before the behaviour begins?	If yes, what comes before?	☐	☐
6. Does any event or behaviour routinely occur immediately after the behaviour begins?	If yes, what follows?	☐	☐

	Yes	No	Unknown
7. Has the dog's general behaviour changed in any way since the onset of the atypical behavior (i.e., the dog is more or less aloof, aggressive, withdrawn, playful, etc.)?	If yes, how?	☐	☐
8. Has the dog's diet recently been changed?	If yes, how?	☐	☐
9. Has this dog seen any of the following specialists for this behaviour?<br><br>☐ Neurologist<br>☐ Surgeon (orthopedic)<br>☐ Internal Medicine Specialist<br>☐ Behavioural Medicine Specialist<br>☐ Dermatologist			

You are done unless your dog is 8 years or older. If they are this age, there is one questionnaire remaining. Thank you!

F. ***Age Associated Changes***

Please complete this questionnaire ***ONLY*** if the dog is 8 years and older.

This questionnaire is about behavior and physical changes associated with aging. This questionnaire was adapted from a series of veterinary medical questionnaires to assess changes in physical and behavioral states and includes modified questions from: Rofina, J.E., Van Ederen, A.M., Toussaint, M.J.M., Secreve, M., Van, D.S., Van, D.M., I, Van Eerdenburg, F.J.C.M., Gruys, E., 2006. Cognitive disturbances in old dogs suffering from the canine counterpart of Alzheimer's disease. Brain Res. 1069, 216-226. Salvin, H.E., McGreevy, P.D., Sachdev, P.S., Valenzuela, M.J., 2011b. The canine cognitive dysfunction rating scale (CCDR): A data-driven and ecologically relevant assessment tool. Vet. J. 188, 331-336. Overall, KL. Manual of Clinical Behavioral Medicine for Dogs and Cats. Elsevier, 2013.

Behaviour screen for age associated changes:

1. Locomotory/ambulatory assessment (**tick only 1**)

- ☐ No alterations or debilities noted
- ☐ Modest slowness associated with aging from youth to adult
- ☐ Moderate slowness associated with aging
- ☐ Moderate slowness associated with aging plus alteration or debility in gait (e.g., limps, occasionally trips)
- ☐ Moderate slowness associated with aging plus some loss of function (e.g., cannot climb stairs)
- ☐ Severe slowness associated with extreme loss of function, particularly on slick surfaces (may need to be carried or need a support harness)
- ☐ Severe slowness, extreme loss of function, and decreased willingness or interest in locomoting (spends most of time in bed)
- ☐ Paralyzed or refuses to move

2. Appetite assessment (**may tick more than 1**)

- ☐ No alterations in appetite
- ☐ Change in ability to physically handle food
- ☐ Change in ability to retain food (vomits or regurgitates)
- ☐ Change in ability to find food when offered, dropped or in dish
- ☐ Change in interest in food (may be olfactory, having to do with the ability to smell)
- ☐ Change in rate of eating
- ☐ Change in completion of eating
- ☐ Change in timing of eating
- ☐ Change in preferred textures

3. Assessment of elimination function (**tick only 1 in each category**)

Changes in frequencies and "accidents"

- ☐ No change in frequency and ***no*** "accidents"
- ☐ Increased frequency, ***no*** "accidents"
- ☐ Decreased frequency, ***no*** "accidents"
- ☐ Increased frequency ***with*** "accidents"
- ☐ Decreased frequency ***with*** "accidents"
- ☐ No change in frequency***, with*** "accidents"

Bladder control

- ☐ Leaks urine when asleep, only
- ☐ Leaks urine when awake, only
- ☐ Leaks urine when awake or asleep

☐ Full-stream, uncontrolled urination when asleep, only
☐ Full-stream, uncontrolled urination when awake, only
☐ Full-stream, uncontrolled urination when awake or asleep
☐ No leakage or uncontrolled urination, but urinates in inappropriate or undesirable location
☐ No change in urination control or behaviour

Bowel control – please select the appropriate answer for the description you choose
☐ Defecates when asleep
  ☐ Formed stool ☐ Diarrhea ☐ Mixed
☐ Defecates without apparent awareness
  ☐ Formed stool ☐ Diarrhea ☐ Mixed
☐ Defecates when awake and aware of action, but in inappropriate or undesirable locations
  ☐ Formed stool ☐ Diarrhea ☐ Mixed
☐ No change in bowel control

4. Visual acuity - how well does the client think the dog sees? (**tick only 1**)

☐ Some change in acuity dependent on ambient light condition
☐ Some change in acuity ***not*** dependent on ambient light condition
☐ Extreme change in acuity dependent on ambient light conditions
☐ Extreme change in acuity ***not*** dependent on ambient light condition
☐ Blind

5. Auditory acuity – how well does the client think the dog hears (**tick only 1**)

☐ No apparent change in auditory acuity
☐ Some decrement in hearing – not responding to sounds to which the dog used to respond
☐ Extreme decrement in hearing – have to make sure the dog is paying attention or repeat signals or go get the dog when called
☐ Deaf – no response to sounds of any kind

6. Play interactions - if the dog plays with **toys** (other pets are addressed later), which situation best describes that play? (tick **only** 1)

☐ No change in play with toys
☐ Slightly decreased interest in toys, only
☐ Slightly decreased ability to play with toys, only
☐ Slightly decreased interest and ability to play with toys
☐ Extreme decreased interest in toys, only
☐ Extreme decreased ability to play with toys, only
☐ Extreme decreased interest and ability to play with toys
☐ This dog has never played with toys

7. Interactions with humans - which situation best describes that interaction? (**tick only 1**)

☐ No change in interaction with people
☐ Recognizes people but slightly decreased frequency of interaction
☐ Recognizes people but greatly decreased frequency of interaction
☐ Withdrawal but recognizes people
☐ Does not recognize people
☐ This dog has never really interacted with people

8. Interactions with other pets - which situation best describes that interaction? (**tick only 1**)

☐ No change in interaction with other pets
☐ Recognizes other pets but slightly decreased frequency of interaction
☐ Recognizes other pets but greatly decreased frequency of interaction
☐ Withdrawal but recognizes other pets
☐ Does not recognize other pets
☐ No other pets or animal companions in house or social environment
☐ This dog has never really interacted with other dogs or cats

9. Changes in sleep / wake cycle (**tick only 1**)

☐ No changes in sleep patterns
☐ Sleeps more in day, only
☐ Some change - awakens at night and sleeps more in day
☐ Much change - profoundly erratic nighttime and daytime sleep patterns with lots of nighttime activity or restlessness
☐ Sleeps virtually all day, awake occasionally at night
☐ Sleeps almost around the clock

10. How often does your dog pace up and down, walk in circles and/or wander with no direction or purpose?

☐ Never
☐ Once a month
☐ Once a week
☐ Once a day
☐ More than once a day

11. How often does your dog stare blankly at the walls or floor?

☐ Never
☐ Once a month
☐ Once a week
☐ Once a day
☐ More than once a day

12. How often does your dog get stuck behind objects and is unable to get around?

☐ Never
☐ Once a month
☐ Once a week
☐ Once a day
☐ More than once a day

13. How often does your dog fail to recognize familiar people or pets?

☐ Never
☐ Once a month
☐ Once a week
☐ Once a day
☐ More than once a day

14. How often does your dog walk into walls or doors?

- ☐ Never
- ☐ Once a month
- ☐ Once a week
- ☐ Once a day
- ☐ More than once a day

15. How often does your dog walk away from or avoid being petted or other loving attention that they have been known to enjoy?

    - ☐ Never
    - ☐ Once a month
    - ☐ Once a week
    - ☐ Once a day
    - ☐ More than once a day

16. Has your dog changed in the way he/she behaves with humans, dogs or cats? Please tell us about any changes. Things in which we are especially interested are a change (increase or decrease – please tell us which) in avoidance, aggression, fear, withdrawal, approach or any other component of social interaction.

17. Is there anything else you think we should know? If you think you have observed something interesting – even if you don't understand it – please tell us.

Thank you so much! We could not do this study without you!
