# Supplementary material for: Effects of Changing Veterinary Handling Techniques on Canine Behaviour and Physiology Part 1: Physiological Measurements
Source: Animals (Basel). 2023 Apr 4;13(7):1253. doi: 10.3390/ani13071253 (PMC10093362; doi:10.3390/ani13071253)
Supplement: Supplementary file 1 [file animals-13-01253-s001.zip › Figure S2 - Homework for intervention group.pdf]

**Figure S2: Client tick sheet for at home exercises with the dog (Homework) – Group 2**

Dog Name______________________________ Client name_____________________________

Veterinary exams require extensive handling. You can help your dog get used to such petting at home.

***For 5 minutes 3 times a week*** please simply sit or lie down with your dog and pet him or her from nose to tail. We need for you to handle all the areas that we will handle in a veterinary exam. We have made a short video to help you with this homework.

1. Use long, slow strokes, similar to a massage, and ensure that you run your hands firmly, but without grabbing over the entire dog, including down all 4 of the dog's legs. If the dog goes to sleep, this is fine. Please ensure that you touch the back of the neck, both sides of the chest and abdomen, the ears and legs.

2. Place your hand firmly on either side of the chest. You should be able to feel the heart.

3. Place your hand on the hips and rump and run it down each hind leg.

4. Place your hands gently but firmly on either side of the abdomen. You can rub or massage gently.

5. We would also like for you to lure the dog – using a treat – to turn his or her head first to the right and then to the left. Handle each ear as it comes closer to you (and if your dog is floppy eared pick up the ear).

6. Again using a treat, pick up for 2-5 seconds and gently place back on the ground each of the 4 feet. The feet just have to come a small amount off the ground.

7. Take a tube of cream or lotion and put some in your hand. The stroke it onto the dog's front parts of their front legs and the sides of the back legs over the hock/ankle. See the video for how to do this.

We have provided a log for you record when you did this and whether you have any comments about the dog's behaviour while being petted/massaged or afterwards.

| Session | Date | Time started petting | Time stopped petting | Comments/concerns/thoughts? |
|---|---|---|---|---|
| 1 | | | | |
| 2 | | | | |
| 3 | | | | |
| 4 | | | | |
| 5 | | | | |

| | | | | |
|---|---|---|---|---|
| 6 | | | | |
