# Supplementary material for: Effects of Changing Veterinary Handling Techniques on Canine Behaviour and Physiology Part 1: Physiological Measurements
Source: Animals (Basel). 2023 Apr 4;13(7):1253. doi: 10.3390/ani13071253 (PMC10093362; doi:10.3390/ani13071253)
Supplement: Supplementary file 1 [file animals-13-01253-s001.zip › Figure S3 - Homework for control group.pdf]

Figure S3: Client tick sheet for at home exercises with the dog (Homework) – Group I

Dog Name_______________________________ Client name______________________________

Veterinary exams require extensive handling. You can help your dog get used to such petting at home.

For 5 minutes 3 times a week please simply sit or lie down with your dog and pet him or her from nose to tail.

- Use long, slow strokes, similar to a massage, and ensure you run you hands firmly, stroke slowly but with some pressure from the neck all the way down to the tail.
- Gently feel/massage the belly and chest on both sides.
- Gently but firmly stroke the inside of the back legs.
- Run your hands down all 4 legs.
- Gently hold and pet the head and ears.

If the dog goes to sleep, this is fine. Please ensure in your petting that you touch the back of the neck, both sides of the chest, the hips and rump, both sides of the abdomen and all legs. We have made a short video clip that will help.

We have provided a log for you record when you did this and whether you have any comments about the dog's behaviour while being petted or afterwards.

Session	Date	Time started petting	Time stopped petting	Comments/concerns/thoughts?
1				
2				
3				
4				
5				
6				
