# Supplementary material for: Effects of Changing Veterinary Handling Techniques on Canine Behaviour and Physiology Part 1: Physiological Measurements
Source: Animals (Basel). 2023 Apr 4;13(7):1253. doi: 10.3390/ani13071253 (PMC10093362; doi:10.3390/ani13071253)
Supplement: Supplementary file 1 [file animals-13-01253-s001.zip › Table S1 - Likert scale.pdf]

Time and tick sheet for each client visit:

Date: ________

Client:____

Dog: ____

People present:____

Time	Activity	Assessment<br><br>scale	Score
	Greet client in parking lot	N/A	
	Entering AVC front door	1	1 2 3 4 5
	Steps on scale	2	1 2 3 4 5
	Entering exam room	3	1 2 3 4 5
	Beginning of exam	4a and from video	1 2 3 4 5
	Draw blood	From video	
	End of exam	4b and from video	1 2 3 4 5
	Treats at end		☐ Took and ate<br>☐ Took and spit out<br>☐ Ignored
	Treats in parking lot		☐ Took and ate<br>☐ Took and spit out<br>☐ Ignored

Scored assessments:

<u>The scoring rubric:</u> The total score possible for scales one to four is 51. Dogs with a zero score are very happy to go to the vet. Dogs with high scores need help. A note on the scoring system: There are now a number of scales commonly used to score dogs and cats undergoing veterinary care. Some scales combine categories 2 and 3 but if the difference can be discerned, it's useful.

In the system here:

- **Dogs with a 0** score are happy-go-lucky and enthusiastic,
- **Dogs with a 1** are truly calm but not enthusiastic (exuberant enthusiasm can actually interfere with an examination).
- **Dogs with a 2** are wary – they are alert and watchful to all changes and will comply but they are not calm as are dogs with a score of 1.
    - It is at the point of wariness – a score of 2 here - that we should begin to attend to what the dog is communicating about their concern and distress.
- **Dogs with a score of 3** are more than alert – they are physically tense and panting – signs of distress – and their tail is likely down.
    - **Dogs with scores of 3** or higher will invariably learn that the procedure was scary and become more averse with exposure.
- **For any dog scoring a 4 or 5** the clinician should consider aborting the procedure and either trying again in a calmer circumstance or with anti-anxiety medication on board.

<u>Clinic Dog Stress Scale 1: Entry to the Clinic</u>

Dog's behaviour upon entering the veterinary practice and in the waiting room (this section can be completed by a member of the reception staff). A total of 5 points are possible. Dogs with a score of 5 are distressed and need help. Dogs with zero-1 scores are calm.

Stress Level	Dog's Behaviour/Demeanour
0	Extremely friendly, outgoing, solicitous of attention
1	Calm, relaxed, seemingly unmoved
2	Alert, but calm and cooperative
3	Tense, but cooperative, panting slowly, not very relaxed but can still be easily led on lead
4	Very tense, anxious, may be shaking or whining, will not sit or lie down if exposed may do so behind owners' legs), panting, difficult to maneuver on lead
5	Extremely stressed, barking/howling, tries to hide, needs to be lifted up or forced to move

Clinic Dog Stress Scale 2: Weighing the Dog

Dog's behaviour upon being weighed (this section can be completed by the veterinary nurse or technician who weighs the dog). A total of 5 points are possible. Dogs with a score of 5 are distressed and need help. Dogs with zero-1 scores are calm.

Stress Level	Dog's Behaviour/Demeanour
0	Extremely friendly, outgoing, solicitous of attention, eagerly gets onto scale
1	Calm, relaxed, seemingly unmoved, and walks easily onto scale and sits
2	Alert, but calm and cooperative, can get onto scale but may not sit on it
3	Tense, but cooperative, panting slowly, not very relaxed but can still be easily led on lead, gets onto scale only with encouragement
4	Very tense, anxious, may be shaking or whining, will not sit or lie down if exposed (may do so behind owners' legs), panting, difficult to maneuver on lead, must be helped/encouraged to get on or stay on scale for 10 seconds to get reading
5	Extremely stressed, barking/howling, tries to hide, needs to be lifted up or forced to get onto or stay on scale for 10 seconds to get reading

Clinic Dog Stress Scale 3: Entering the Exam Room

Dog's behaviour upon being brought into the exam room (this can be completed by whomever guides the client and dog to the room). A total of 5 points are possible. Dogs with a score of 5 are distressed and need help. Dogs with zero-1 scores are calm.

Stress Level	Dog's Behaviour/Demeanour
0	Extremely friendly, outgoing, solicitous of attention
1	Calm, relaxed, seemingly unmoved
2	Alert, but calm and cooperative
3	Tense, but cooperative, panting slowly, not very relaxed but can still be easily led on lead
4	Very tense, anxious, may be shaking or whining, will not sit or lie down if exposed may do so behind owners' legs), panting, difficult to maneuver on lead, avoids room
5	Extremely stressed, barking/howling, tries to hide, needs to be lifted up or forced to move into room

Clinic Dog Stress Scale 4a: The beginning and the end of the exam

Dog's behaviour upon being brought into the exam room (this can be completed by whomever guides the client and dog to the room). A total of 5 points are possible. Dogs with a score of 5 are distressed and need help. Dogs with zero-1 scores are calm.

**Stress Level**	**Dog's Behaviour/Demeanour**
0	Extremely friendly, outgoing, solicitous of attention
1	Calm, relaxed, seemingly unmoved
2	Alert, but calm and cooperative
3	Tense, but cooperative, panting slowly, not very relaxed but can still be easily manipulated for exam and cooperates with procedures
4	Very tense, anxious, may be shaking, whining or frozen, difficult to maneuver, tries to avoid exam, may hold onto table but not cooperate – more endures
5	Extremely stressed, barking/howling, tries to hide, needs to be lifted up or held to be examined or would bolt from table or room, difficult to control and may be tempted to bite, may not be able to complete exam.
