# Supplementary figures and images for: Effects of Changing Veterinary Handling Techniques on Canine Behaviour and Physiology Part 1: Physiological Measurements
Source: Animals (Basel). 2023 Apr 4;13(7):1253. doi: 10.3390/ani13071253 (PMC10093362; doi:10.3390/ani13071253)

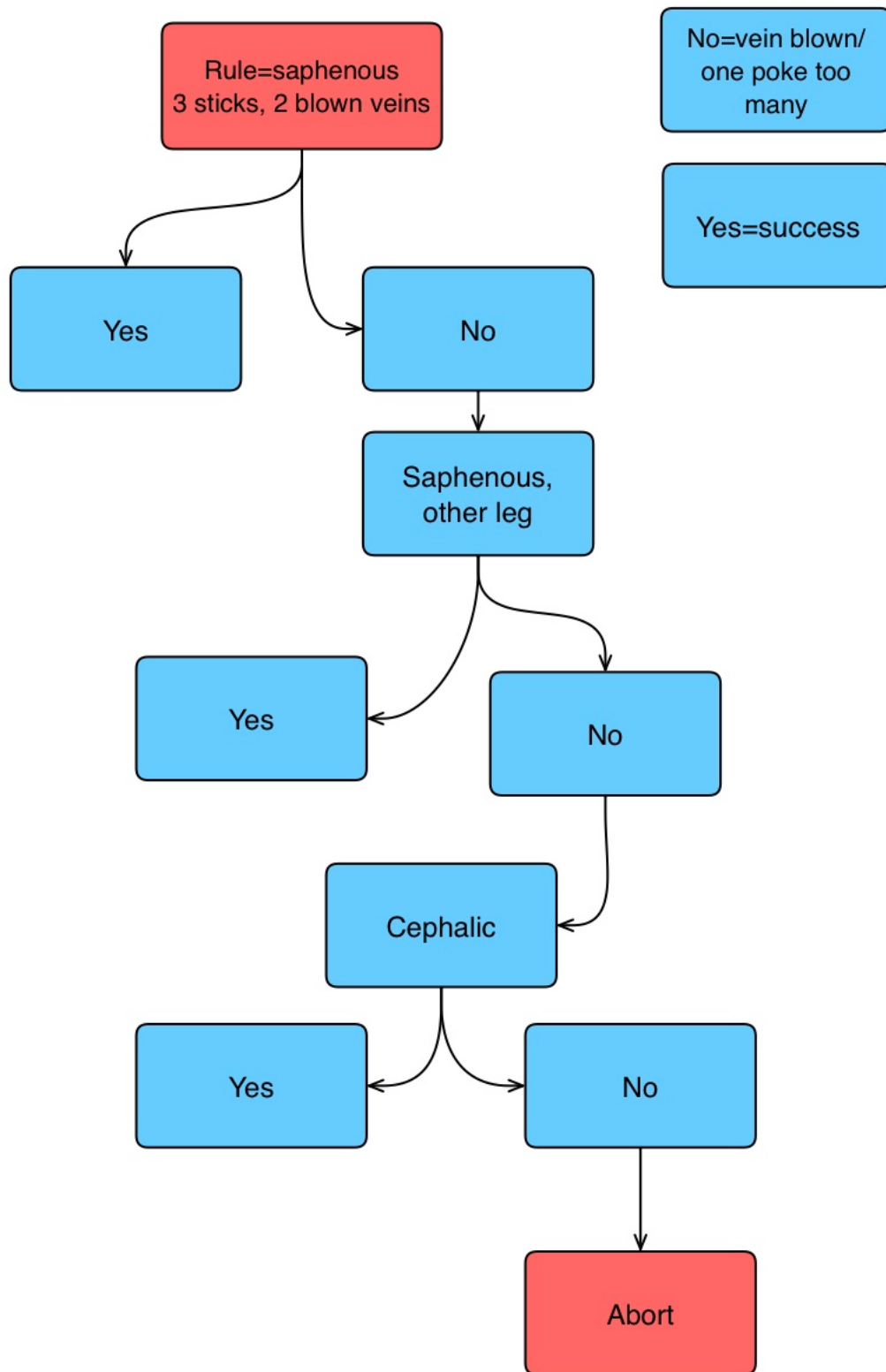

Supplement: Supplementary file 1 [file animals-13-01253-s001.zip › Figure S4 - Blood draw decision tree.pdf]

No=pet distressed/  
restraint not  
working

Yes=success

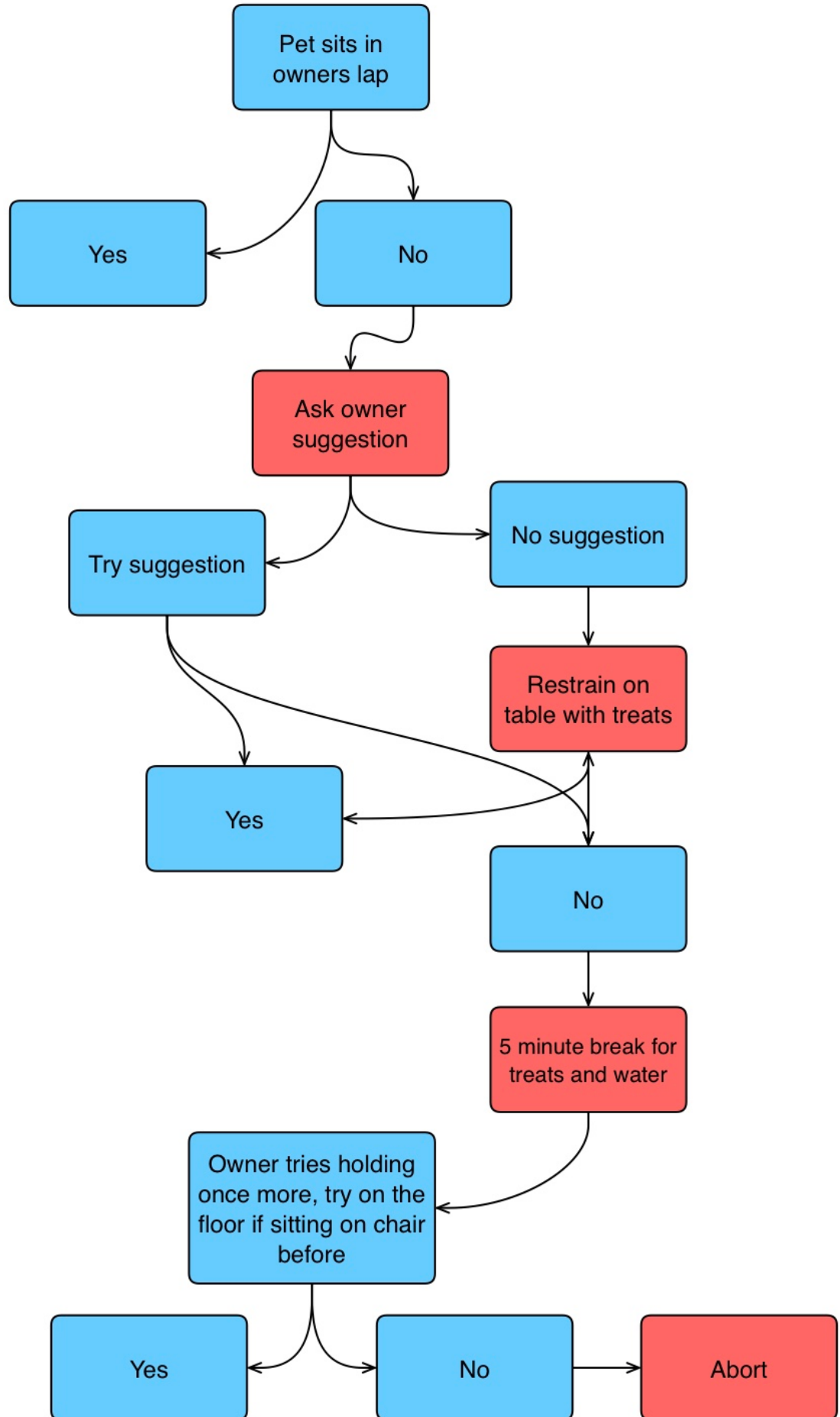

Supplement: Supplementary file 1 [file animals-13-01253-s001.zip › Figure S5 - Restraint decision tree.pdf]
